# The Experiences and Expectations of Older Adults and Close Family in Nursing Home and Emergency Department Transitions: A Qualitative Study

**DOI:** 10.1155/jare/8896040

**Published:** 2025-10-15

**Authors:** Elin Høyvik, Malcolm Bray Doupe, Gudmund Ågotnes, Frode Fadnes Jacobsen

**Affiliations:** ^1^Centre for Care Research, Western Norway University of Applied Sciences, Årstadveien 17, Bergen 5009, Norway; ^2^Max Rady College of Medicine, University of Manitoba, 727 McDermot Avenue, Winnipeg MB R3E 3P5, Canada; ^3^Department of Welfare and Participation, Western Norway University of Applied Sciences, Inndalsveien 28, Bergen 5063, Norway

**Keywords:** emergency department, nursing home, resident, transition

## Abstract

**Aim:**

To identify the experiences, expectations, and preferred transitional care expressed by nursing home residents and close family, thus mapping perceived barriers and facilitators to improve this identification process.

**Design:**

In this study, a qualitative design was employed.

**Methods:**

Individual, semistructured interviews were conducted.

**Data Sources:**

Interviews of 12 participants (3 residents and 9 close family) were conducted. The data were analyzed using thematic analysis to identify underlying themes.

**Results:**

The following three themes were identified: (1) changes in life situations, (2) dimensions of transfer quality, and (3) interactions with staff.

**Conclusion:**

Nursing home residents and close family emphasize that proper medical care is necessary. However, this is insufficient without addressing multiple ongoing life changes of individuals transitioning between nursing homes and emergency departments. Yet, this effort to manage life changes is significantly insufficient without the support of healthcare professionals.

**Summary**

• Implications for practice
◦ This study emphasizes the need for a comprehensive approach to transitions, recognizing life changes and individual circumstances.
◦ It highlights the importance of compassionate interactions and tailored support for nursing home residents and their family members to enhance their experiences.
◦ In addition, the study calls for further research and policy efforts to address systemic barriers in transitions and ensure healthy transitions for individuals.
• Impact
◦ This study outlines barriers and gaps in care delivery during transitions between nursing homes and emergency departments and provides useful information for developing and field-testing interventions.
• Reporting method
◦ This study adheres to the Consolidated Criteria for Reporting Qualitative Research (COREQ) guidelines.
• Patient contribution
◦ Nursing home residents and close family were interviewed for this study.

## 1. Introduction

Nursing home (NH) residents frequently use emergency healthcare services [1]. In particular, older adults are identified as vulnerable during their transitions between healthcare service organizations [2], and they often enter emergency departments with multiple health conditions and significant healthcare requirements [3]. Transition, by definition, includes *“a passage from one life phase, condition, or status to another, is a multiple concept embracing the elements of process, time span, and perception”* ([4], p. 239). In this study, transitions include events that take place before, during, and after the physical transfer.

NH⇄ED transitions represent a critical part of geriatric healthcare. Therefore, addressing the complexities in transitional care becomes imperative when improving the health and well-being of older adults in NH⇄ED transitions. This study identifies preferred strategies and areas that need improvement from the perspectives of NH residents and close family (CF) during the NH⇄ED transition.

## 2. Background

NH residents transferred to the ED are at risk of physical ailments or increased cognitive decline. Unclear reasons for transfer, combined with the complex needs of older adults, often result in inappropriate care [5]. Many NH⇄ED transitions may not be beneficial to patients and can even worsen the health situation of frail older adults with increased risk of delirium, infections, falls, functional decline, and mortality [6]. Furthermore, the negative outcomes of transitional care have been linked to discharge at unfavorable hours [7]. Other factors contributing to the dissatisfaction of older adults and CF in transitions have also been identified, such as lack of information [8], poor relationships between caregivers and patients or their family [9], insufficient family support [10], and inadequate patient participation [11].

In Norway, a growing number of older adults with complex health issues combined with an expected future decline of available staff members have been identified ([12]: 4, p. 12). This challenge has also been identified in other Nordic countries [13]. Norwegian municipalities are expected to provide more advanced healthcare treatment in more recent years, and efficiency requirements prompt hospitals to discharge patients at an earlier stage than previously (Meld. St. 47 [14]). This requires advanced cooperation between different service levels, which has been difficult to achieve, although both hospitals and municipalities seek improved ways of communicating [15]. A recent government white paper identifies the need for more coherent healthcare services during transitions due to the following challenges: lack of planning, communication, flow of information, updated medication lists, and follow-up (Meld. St. 9 [16]). Internationally, many countries lack policies or transitional care interventions targeted at NH residents [17]. Moreover, the interventions often focus on isolated aspects of transitions and do not encompass all stakeholders involved [18]. Experiences from a Norwegian context can contribute valuable insights into an international context, as other countries are facing similar issues raised in this study, such as a growing number of older adults with acute and complex healthcare needs [19] and an anticipated rise in demand for healthcare staff [20]. Information from this study applies to countries aiming to improve primary and specialist healthcare collaboration across organizations to accommodate the acute healthcare needs of NH residents.

In this study, transition theory was applied, as articulated by Schumacher and Meleis [21]. This theoretical framework provides a comprehensive understanding of the transition concept, acknowledges the complexity of transitions, and looks beyond the patient transfer from one place to another. Furthermore, this framework recognizes that being in transition is a complex and dynamic process that entails personal, social, and cultural factors [21]. The theory has a normative dimension in identifying the factors contributing to healthy transitions: adapting to changes in roles, relationships, routines, or conditions. Healthy transitions bring positive outcomes and enhance well-being, whereas unhealthy transitions constitute the opposite [22]. Accordingly, the theory can help identify critical areas that need improvement by assessing preferred transitional care.

As individuals undergo NH⇄ED transitions, it is significant to explore their needs and experiences in transitional care [23] before developing interventions customized for NH residents. However, there is a lack of insight into NH residents and CF perspectives during NH⇄ED transitions [24], and little is known about individual outcome goals in these processes. [25]. To improve transitional care between NH and ED, it is crucial to understand the unique experiences of those involved throughout the transitions. Drawing from transition theory's emphasis on how factors like interpersonal support and role changes shape individual adaptations to health-related transitions, this study asks the following: *What are the experiences and expectations of older adults and close family during NH⇄ED transitions?* The study aims to identify the aspects of transitions preferred or disfavored by NH residents and CF, thus recognizing what is important to them during NH⇄ED transitions. The purpose of this study is to inform the development and field-testing of targeted interventions for NH residents during these transitions.

## 3. Method

Qualitative descriptive design with individual semistructured interviews was applied in this study to explore the unique experiences of individuals [26, 27]. An open-ended interview guide was developed, guided by knowledge from the existing literature and transitions theory. For example, the framework pinpoints that the exchange of information impacts transitions, and the participants were asked how they received information during the process. Furthermore, questions were designed to encourage the participants to share their experiences without too much guidance from the interviewer. The interview guide was piloted and revised accordingly, aiming for its relevance in the practice field.

The following inclusion criteria were used to identify NH residents and CF: possessing experience with NH⇄ED transitions, able to recall and recount previous experiences, and having the physical and mental capacity to participate. People with mild cognitive impairment were not excluded, as they can potentially look back on relevant occasions in life and are, therefore, important contributors to patient perspectives [28]. NH administrators facilitated a mixed sampling by sharing information about the study with available participants who fit the inclusion criteria and had the competency to consent. This study complies with the Declaration of Helsinki guidelines to protect vulnerable research participants [29], and it is acknowledged that NH residents are considered particularly vulnerable to research participation. Measures were taken to safeguard the participants' dignity and integrity by including only those with the capacity to consent and who were able to recall and recount their experiences, as verified by the NH staff. An information letter was distributed to participants, which stressed that participation was voluntary and that they could withdraw from the study without further explanation. This information was repeated before starting each interview. Validation was also sought from the NH administrators regarding the participants' ability to comprehend, agree to, and withdraw from participating in this study. The interviewer monitored any potential signs of distress during the interviews. Upon completion, the interviewer stopped the audio recording and briefly followed up to assess the resident's well-being. Information about the study and interview questions was adapted and repeated when necessary. Audio recordings were transcribed verbatim and stored in a secure research database. Potentially identifiable information was anonymized.

The first author interviewed 12 participants (3 NH residents and 9 CFs) in the southwestern region of Norway individually during fall 2023 and spring 2024 (Table 1). One participant was interviewed via Zoom, as requested, and the rest were interviewed at their preferred location. CF shared their own experiences but also offered to speak on behalf of the NH residents. NH residents and those represented by CF had both short-term and long-term NH stays for at least 3 months. The NH residents needed around-the-clock care for various reasons, such as Parkinson's disease, dementia, stroke, pulmonary disease, and others. Participants represented rural and urban areas and came from larger and smaller NHs in Norway. The total number of participants was considered satisfactory, as the available data were sufficient to achieve the aim of the study and obtain information power [30]. We also observed that the final interviews did not yield any new information or perspectives.

Thematic analysis by Braun and Clarke [31] was conducted to identify themes that focused on data rather than preconceived categories. The analysis of the study facilitated transparency, as the results were documented. Data analysis was done according to the following six steps of thematic analysis: (1) Familiarization: After transcribing interview verbatim, the first author revisited the data multiple times to identify potential patterns. (2) Generating initial codes: Data were reduced and categorized into manageable topics based on common characteristics. (3) Searching for themes: Recurring topics were identified and coded into potential themes using NVIVO software based on the initial categorization. (4) Reviewing themes: Initial themes were critically assessed, reevaluated, adapted, or merged by all authors to ensure that they accurately represented the data. (5) Defining and naming themes: The final themes were discussed and scrutinized until consensus was reached within the research team, ensuring that themes aligned with the content of the interviews. (6) Producing the report: The findings were abstracted to accurately represent and support the selected themes for a coherent and comprehensive narrative. Weaknesses of thematic analysis include potential subjectivity, which was amended by including all authors in the analysis. Furthermore, thematic analysis can be too superficial if one misses complex nuances, a challenge that was addressed by revisiting the data multiple times.

This study was approved by the Norwegian Centre for Research Data (Ref. 640165) and followed the Consolidated Criteria for Reporting Qualitative Research (COREQ) guidelines to ensure transparency and trustworthiness. AI tools like Grammarly and Keeinous have supported this study by improving language, grammar, phrasing, and aiding literature searches. However, due to their limitations in critical analysis, contextual understanding, and reliability, they were used only as supplementary tools rather than shaping the content. This study adheres to the Best Practices Guidelines on Research Integrity and Publishing Ethics.

## 4. Findings

This section presents key findings that pointed toward the experiences and expectations of NHR (NH residents) and CF, and the following themes were identified: changes in life situations, dimensions of transfer quality, and interactions with staff members (Table 2).

### 4.1. Changes in Life Situations

The participants were already undergoing multiple changes when a NH⇄ED transition was initiated, such as becoming a NH resident, declining physical and mental health, and developing dementia. These transitions were still active and unresolved, affecting what they carried into the NH⇄ED transition regarding their needs, resources, and adaptability.

From being healthy and self-sufficient individuals, older adults had become dependent on continuous healthcare. The participants in this study lamented the process of declining health, which at times felt unmanageable. Many family members shared how observing the changes in the health status and loss of independence in their loved ones' lives heightened their concerns about further deterioration. The deteriorating health of NH residents had an impact on their lives, which at times evoked feelings of loss and bereavement: *“We used to travel a lot and had plans for many more”* (CF5). Each hospitalization caused concern to the participants, serving as a reminder of the decline in their health: *“Because every time I get this, I go… decline all the way”* (NHR1). Some participants felt that even though it was painful to have declining health and become acutely ill, NH⇄ED transitions were a normal part of aging: “*You are 1 year older, and suddenly you are sick. That's when you are standing on the threshold for real. How much more powerful it is when you have some sort of physical or mental illness? Like: ‘Oh well, you are so old, it's only reasonable*'” (NHR2). Other participants experienced that the NH⇄ED transitions symbolized the end of life for the residents:

“From one moment to the next, your life has turned upside down. You are on the other side. I used to be healthy, and I lived my life to the fullest, and I guess, I pictured I was going to function. And suddenly, that was over, and it was a new era. And you know it's the last one, so you must deal with it” (NHR2).

A decline in health evoked certain emotions in the participants, sometimes to the point of exhaustion, as they were already experiencing a series of transitions before the NH⇄ED transition. Several NH residents had several hospitalizations and short-term NH stays without being able to settle down. Becoming an NH resident was a significant transition for the participants of this study, as it meant leaving parts of your life behind: *“It's a transition for everyone. Break up with your home when you feel fairly well. It's a huge process. So, I have to say it took a mental toll on me. It did. And I had to do this immediately, so I felt I wasn's a part of the process with my things. But I have put it aside that they are dead things, and I have what I need”* (NHR2). In addition, becoming an NH resident also meant parting with life as they knew it: *“It is basically a settlement with life. You kind of feel that you say good-bye to life in a way. At the same time, you are still there”* (NHR2). Participants also highlighted that transitioning to the NH brought about a sense of grief, as it signified a change in relationships and their way of life: *“She had to go to a nursing home. It was a sad day. It was bad. We had been together for many years when this happened; we got together in 1976. We always had an enormous amount of fun together; it was horrible”* (CF4). The transition to NH also meant mourning the loss of something familiar, such as parting with their residence.

For CF participants, the aforementioned life changes had an added dimension. Not only did the life changes in the older adults affect their own lives but also prompted shifts in their roles and relationships with the NH residents. For most of the CF participants, this resulted in increased responsibility as caregivers: *“Eventually, as the illness progresses, there is a gradual change. Considering she is not able to answer on her behalf, you as close family are, as her guardian, assigned to take care of her well-being”* (CF9). The CF members reflected on their role change, and some participants felt overwhelmed by the increased burden as caregivers: “*The kids saw I struggled. I wore myself out because he was heavy*” (CF5). Being present as a CF member during a NH⇄ED transition sometimes included a sense of fear: “*My biggest fear is that when they call me it's the end for my wife, and every time she goes to the hospital is a harsh reminder that we're getting close to that point*” (CF1). Another issue brought forth by CF was the experience of witnessing the illness of their loved ones:

“It was a horrible sight to come here. She laid there and could hardly breathe. She gurgled immensely; it was as if she had a liter of water in her lungs and couldn't breathe. She had… it was the worst sight of my life, ever” (CF4).

However, not all CF participants with increased caregiving responsibilities viewed this to be a burden, but also a as a necessity. For example, one daughter arrived at the ED to find her delirious mother unclothed, and the staff member appeared overwhelmed. She ended up taking over, helped calm her mother down, and got her dressed. Another participant appreciated being able to support the NH resident: “*But I ended up taking responsibility for certain things. I didn't see it as a burden. To be honest, it has been my way to help and support*” (CF9). Eventually, some participants came to terms with the life changes that were present at the time of the NH⇄ED transition, which included a sense of acceptance that things were changing.

### 4.2. Dimensions of Transfer Quality

Many participants thought the healthcare professionals did a good job providing medical treatment, which made them feel appreciated: “*So when they address this immediately, you feel appreciated, it is sort of like they want me to stick around for a little longer, so to speak”* (NHR1). Most participants trusted the staff members and their competence. However, some participants said they were unsure if the medical treatment held the necessary standards, as they did not know how to assess that. Therefore, they had to put faith in healthcare professionals. Furthermore, in many situations, participants questioned whether they received the required treatment. As a result, they felt the illness went unmanaged, often disrupting the recovery. Some participants shared that medications were not distributed as planned, resulting in prolonged health issues. The participants not only experienced a lack of or wrongful medical treatment but also felt the care itself could be detrimental to the residents: “*They [staff] grabbed the little pink sheet, and it was full speed from both sides. He became hysterical, raised his fist, hysterical. And then he's like, they are moving too fast, without letting him know they are about to turn him*” (NHR3, describing the period when her husband was acutely ill). The experiences of not receiving proper medical healthcare could, for some participants, limit the trust they had in the staff members, as they found it difficult to have faith in the quality of the care provided: *“I think it's scarier now if he is admitted to the hospital. I feel that I have lost a little trust. Not that I think they won't cure him, but I have also experienced that they return him home too soon*” (CF6).

Participants expressed that, at times, residents did not have their basic needs met, including personal hygiene, nutrition, hydration, toileting, and sleep, to mention a few: “*But she didn't receive personal hygiene care until nighttime. That would be afternoon, evening*” (CF3). Sometimes, NH residents were placed in the hallway, which was bothersome to them due to light and noise. Furthermore, residents also had to wait to go to the toilet or eat. The wait during NH⇄ED transitions was difficult for participants, which often went without addressing their necessities. Sometimes repeated pleas for help would be ignored, according to participants: “*My husband shouts that he must go to the restroom: “You need to come, I'm about to soil my pants,” like that. He does, and then it is: “I need to go to the restroom again” [NH resident]. “You just went” [ED staff]* (NHR3, describing the period when her husband was acutely ill).

Neglected needs often led participants to tend for themselves during the NH⇄ED transition, which made them feel ignored and isolated. In similar situations, CF participants would take on the tasks of healthcare professionals when they felt that this was needed. This could mean assisting them with toiletry, feeding, and other things. This was rather common when the resident had dementia or delirium, in which case the family member would assist in comforting the NH resident if he or she was confused and distressed. Furthermore, CF would search for a healthcare staff member to ask for assistance when they were unavailable: “*He laid in the hallway, and he had a really dry mouth. So, I ran around: Can I get help? What do I do?”* (CF6). Several CF participants also shared that their own needs were unmet. For example, one participant explained that he did not eat for 12 h after arriving at the ED. Many CF participants would downplay their own needs during NH⇄ED transitions, as they would acknowledge the pressure the healthcare staff experienced and accept the urgency of the situation. CF members did not want to add to the stress, as they felt that they were not the focus of attention of the staff. Their needs often had less priority, so they sometimes refrained from contacting healthcare professionals for help to address their discomfort.

According to the participants, long waiting hours impacted the well-being and safety of the residents, which could end up in incidents that went unnoticed: “*He was all alone. If he was in pain, he couldn't call for anyone, and he had to wait until they came*” (CF6). Moreover, extensive waiting hours while being ignored by the healthcare staff sometimes contributed to feelings of hardship: “*It was pretty rough. You lie, and you lie, and you lie, and you lie. And getting to the restroom was awful. And then there was the bed. Yeah, that was not a good experience*” (NHR2).

Another challenge affecting the experience of the participants in this study was uncertainty about the next steps. Participants were often unaware of the plan or progress in their NH⇄ED transitions and were not automatically updated. It was not uncommon for participants to acquire this information independently by actively seeking answers from healthcare staff. Participants often felt they would not receive enough information unless they specifically asked for it. One CF member even felt that her efforts directly improved the outcome of the NH⇄ED transition: “*I feel that a lot of the information I have received and help he has gotten has been because I have asked for it (…). Probably very persistent for many, but I don't think he would be as well had he not had me doing what I have done”* (CF6).

Some NH⇄ED transitions experienced by participants were quite upsetting, which left a lasting impression on them. Being informed of the process was of great value in these situations, as it helped them address the situation. When relevant information was missing, they experienced feelings of hopelessness:

“It got very extensive, very quickly. I wasn't ready. But when I saw him the way I saw him, I wasn't ready for the fact that he might not be here tomorrow when I get here. But there wasn't anyone who took the initiative to that conversation; I did the digging. I wanted an honest response immediately to be prepared” (CF6).

For CF participants to residents with dementia, being informed was important for their ability to support the residents. Underinformed CF participants were at times frustrated, as they felt confused and distressed for being unable to support their loved ones properly. Examples were also provided where staff members did not provide the necessary help they asked for, which could be confusing for CF and NH residents. At times, participants reported that healthcare professionals could not access relevant patient information. This concerned them, and some would question why healthcare professionals had not already obtained this information. At times, healthcare staff did not even know the resident's location.

### 4.3. Interactions With Staff

Interviews showed that interactions with healthcare professionals significantly impacted patients' and CF members' overall experiences during NH⇄ED transitions. The participants repeatedly emphasized the value of nurturing, empathetic, and supportive interactions with staff. These encounters with staff members could result in feelings of a positive NH⇄ED transition despite the shortcomings in medical treatment. In contrast, participants also described dismissive behavior from healthcare professionals, which could destroy an otherwise smooth NH⇄ED transition from a medical perspective.

Being heard by the staff members was vital for the participants. This made them feel valued and understood, as they also felt included in the process: “*Be there for us and listen to us. It is basically very important that you feel you're being heard, what we say and want*” (NHR2). Some participants stressed the importance of communication between residents, CF, and healthcare professionals.

The participants of this study highlighted the impact a warm and empathetic attitude from a healthcare professional had on the overall experience of the NH⇄ED transition. When healthcare professionals displayed a warm and nurturing attitude, residents and CF often grew fond of those staff members. Positive encounters with healthcare professionals had a significant impact on the NH⇄ED transitions, as it made the overall experience much easier: “*And she was really sweet, she connected with my mom (…). Even though it was like in the middle of the night, she took the time to check that she was mentally present*” (CF7). A compassionate attitude was significant to the experiences of NH⇄ED transitions according to the participants, as it meant that staff members were attuned to the well-being of the residents. Furthermore, participants appreciated interactions in which they were treated as equals. For some, that would mean being treated in the same way as in any other aspect of life.:

“That they can take me as I am and treat me like a human being, as I am. Normal. The way you and I treat each other, so to speak. Not worse than that. You know, you wouldn't accept someone not being nice to you.” (NHR1).

Being heard and taken seriously was also important to the participants, which included having your inputs considered:

“And I have to say, in the middle of all, I find it positive, both here and in the hospital because I am taken seriously. When you are in pain all the time, you worry that they only think you are whining. But things are being taken seriously.” (NHR2).

During the NH⇄ED transitions, small acts of kindness were crucial. Smaller things helped residents during acute illness: “*They [staff] transferred me [NH resident] into a new bed, and she [nurse] fixed and asked if I was comfortable and so on and if my foot hurt and looked after it, and yeah. And showed care, basically. Very good care*” (NHR3). Many times, these actions were not necessarily related to medical care, as participants valued when staff members took the time to communicate. Participants acknowledged that staff members were preoccupied, but they found comfort in small encounters with the healthcare professionals: “*He speaks highly of those who take the time to talk to him, he thinks it's important. And it's not about them… He knows they don't have the time to sit all afternoon and drink coffee, but it usually takes five minutes or two minutes (…). He finds that important, just to be a little social*” (CF6).

The encounters with ED staff were described as different from those with the NH staff. Participants would describe a faster-paced environment at the ED in which they did not know the staff members and vice versa. However, other factors could foster trustworthy relationships in the ED, such as staff members spending time with the residents: “*It's about the care they show toward the patients. They are busy at the nursing home, too, right? However, they are not as busy, for example, at the ED and the hospital. It's the little things that make them have time to show a little compassion, so to speak. A little more care*” (CF4).

Moreover, participants also shared accounts of meetings in which staff members' behavior negatively impacted the experiences in NH⇄ED transitions. Some participants mentioned rather dismissive encounters with healthcare professionals: “*What I remember specifically about him is that he seemed to be in an awful rush, I would say. It wasn't easy getting anything out of him, you can say. He came in the door and left as soon as he came*” (CF4). In certain instances, some healthcare professionals were perceived as impolite. One resident cried from exhaustion after being in the emergency department for hours and shared this with a staff member:

“And she [staff] said, ‘If I thought I was going to be prioritized when I brought such information, you must understand that it is impossible to suggest such things.' And I was so sad. I was so sad because I didn't ask for that to be taken into consideration” (NHR2).

Such encounters leave a lasting impact on residents and their CF and decrease their overall trust in the healthcare system. Furthermore, it makes participants feel that the staff members did not care for them when their requests were dismissed: “*They sent me astray, and I spent half an hour in the ED before I found him because nobody would help (…). It was very sad and disappointing. I felt nobody cared, that nobody had time*” (CF6). Some participants felt discomfort when they did not receive updates or information from staff members: “*He didn't know anything, understand anything, and was afraid. That was uncomfortable*” (CF6). Meeting staff members who were dismissive toward them could also make them feel helpless:

“He can wait. We'll come when we have time. And we have control” [staff members]. They might have, but as close family, when nobody stops to ask, “Do you need anything? Are you okay?” He has to wait a little. So yeah, when they don't have time to stop, they feel quite helpless” (CF6).

## 5. Discussion

This study adds new insight by revealing how NH residents and CFs experience several transitions at once, often without time to adapt before the next begins. Our data showed that life changes usually serve as a prerequisite to the NH⇄ED transition experience, and they appear to be ongoing health crises rather than isolated events. Given the complex nature of NH⇄ED transitions, it can become difficult for individuals to maintain a sense of stability in the middle of chaos. The existing literature states that one should address prior history when caring for older adults admitted to the ED [32]. Transition theory adds that transitions are connected to previous life experiences [22], which pinpoints the importance of acknowledging the personal history of individuals in transitional care. CF participants also revealed a cumulative burden caused by the life changes of NH residents, beyond the immediate transitions. This indicates that the declining health and closeness to end of life of NH residents can incite feelings of grief and bereavement for CF. Peguero-Rodriguez et al. [32] add that the experiences of NH residents often impact family members, as the latter advocate for their loved ones. We find that the needs of CF members are sometimes overlooked in NH⇄ED transitions, leading to stress and anxiety. Etkind et al. [33] suggest that following the acute illnesses of older adults, there should be a discussion of care preferences while trying to adapt to the current way of living. Our study adds that the simultaneous nature of transitions significantly affects the experience of NH residents and CF in NH⇄ED transitions. Similar to added waves in an already stormy sea, it is difficult for individuals to keep their heads above water. The complexity feels overwhelming in these nested dynamics, as individuals are unable to adapt and settle before the next transition begins. Therefore, recognizing prior life changes and understanding individuals' current life stages during NH⇄ED transitions can be a pivotal approach to transitional care.

Participants of this study described unmet basic needs and discomfort during NH⇄ED transitions, often tied to long waiting hours and inadequate communication. These findings align with other studies reporting extended waiting times and a lack of communication in the ED, which sometimes come at the expense of basic needs [34–36]. Furthermore, it is not uncommon for older adults to experience functional decline after ED visits [37], yet transfers are made without clear evidence of benefits [38]. These findings point toward a particular risk when being involved in NH⇄ED transitions, implying that acute care for older adults holds the potential to add to their ailments. This underscores the relevance of a safe transition pathway, where knowledge sharing and patient involvement can assist healthcare professionals in navigating complex care transitions [39]. Our findings also reveal instances of missing or inaccurate information given to NH residents and their CF members. Prior research states NH residents often receive insufficient information during transitions [2], leading to dissatisfaction among the individuals [40]. Providing information to CF is often ignored in the ED, hindering the role of the latter as caretakers to older adults [32]. Our findings add that NH residents and CF prefer receiving unprompted information, highlighting the importance of providing regular updates. Ultimately, there seems to be a gap in the delivery of care, specifically in addressing the needs and well-being of individuals. Thus, NH⇄ED transitions can potentially add to the discomfort of NH residents and CF.

The participants of this study also highlighted the importance of interactions with staff members during NH⇄ED transitions. Small acts of compassion, such as offering a cup of coffee, carried great significance to those in transition, sometimes to the point of accepting or forgiving the poor or lacking transitional care. In contrast, dismissive or rude behavior could potentially deteriorate the overall experience of the participants despite receiving high-quality treatment. There is evidence that most CF of older adults in EDs report satisfactory interactions with staff, but some report that poor communication and dismissive behavior negatively affected their overall experience [41]. Positive interaction with a healthcare provider has been reported to reduce anxiety, clarify expectations, build trust, and improve the overall experience [35]. Transition theory also identifies that supportive relationships are imperative to transitions, as empathetic and trustful interactions can support individuals while promoting well-being, healing, and improved health outcomes [21]. The framework also emphasizes that positive encounters include support, information, and reassurance, whereas negative interactions can lead to stress and hinder adaptation [42]. This underscores the critical role of staff members' behavior and communication in shaping the experiences in transitions.

Additionally, our findings demonstrate that the needs of CF add an extra dimension, as their emotional well-being directly impact their ability to support their loved ones. Fry et al. [41] state that the presence of CF members is important in supporting older adults and healthcare professionals. Thus, fostering nurturing and supportive encounters with CF can benefit the NH residents, as they can draw strength and comfort from their family members. This study argues that due to the proximity to their loved ones, CF members have an important role to play in helping NH residents navigate the stormy sea, as referenced earlier. Interventions such as standardized transfer forms and checklists combined with web-based communication tools can reduce the emotional strain on individuals [23]. Hwang et al. [43] suggest that transitional care nurses can support NH residents and CF involved in ED transitions. The proposed interventions demonstrate that there are tools available to enhance relationship-building. This study adds that interactions with healthcare professionals can impact the success or failure of NH⇄ED transitions. The participants' emphasis on positive interactions differs significantly from a similar study addressing healthcare professionals' perspectives on transitional care, in which they place far greater importance on medical treatment than interactions with patients and CF [44]. This can potentially challenge care provision, as there seem to be different priorities among staff members, NH residents, and CF. Therefore, this study questions whether a differing understanding of what is highlighted can impede healthy transitions. Furthermore, establishing a unified set of values between staff members, NH residents, and CF can be difficult to execute due to the brief nature of interactions during NH⇄ED transitions.

### 5.1. Limitations

This study does not provide sufficient data to identify potential contrasting opinions between NH residents and their CF. Ninety NH administrators were contacted in total, and few facilitated participation, often due to a lack of eligible or willing candidates. Ultimately, only 3 NH residents were recruited. We would have preferred to interview more NH residents, as they provided rare and valuable insights into patient care perspectives, which are often missing in existing research literature. The participating residents also appeared to possess mental and physical abilities that might surpass those of the average NH resident, such as being more physically independent. They may not have represented the most vulnerable NH residents, but they provided a unique patient perspective that was useful to this study.

### 5.2. Implications for Research and Practice

This study highlights that compassionate interactions with staff members are pivotal to the experiences of NH residents and CF. Healthcare professionals should therefore foster empathetic engagement to accommodate the needs of NH residents and CF. Future efforts should explore ways of alleviating the burden experienced by CF, enabling them to support both NH residents and healthcare professionals. Addressing communication gaps, unmet basic needs, and long waiting times requires both collaboration and structural improvements.

As mentioned previously, NH residents were particularly hard to recruit, and we encourage future research to explore alternative ways to obtain first-hand experiences from a vulnerable demographic. Future research should prioritize the inclusion of vulnerable NH residents while exploring how different priorities between staff members, residents, and CF may affect transitional care outcomes. This study also highlights areas in need of improvement from a NH resident and CF perspective, and research should identify solutions to these specific issues.

## 6. Conclusion

This study reveals that NH⇄ED transitions often involve deficiencies in care, often caused by communication issues, lack of information, and long waits, culminating in unmet basic needs and distress. These challenges extend beyond acute illness, as prior and increased vulnerabilities shape how NH residents and CF experience care. This study highlights that transitions are not isolated events, but interrelated and emotionally challenging. Compassionate and supportive interactions with staff members play a crucial role, while negative interactions can overshadow even high-quality medical care. Addressing these issues requires a comprehensive approach that recognizes prior life changes and the emotional needs of individuals. Healthcare professionals should foster warm and nurturing interactions that ensure that individuals receive information and proper medical treatment. Alleviating the CF burden through better communication and inclusive practices can enhance their ability to provide support during transitions.

## Figures and Tables

**Table 1:** Sample characteristics.

Participant type	Number	Specifications
NH residents	3	3 women
Close family	9	5 women, 4 men
Spouse	3	1 woman, 2 men
Daughter/Son	5	3 women, 2 men
Granddaughter/Grandson	1	1 woman
NH resident age	Range 83–96	Including those represented by close family
Reasons for NH⇄ED transition		
Pulmonary disease	1	
Fall	7	
Infection	4	

**Table 2:** Themes, subthemes, and selected quotes.

Theme	Subtheme	Selected quotes
Changes in life situations	Declining health and functional abilities	“*She started wandering at night. She would hallucinate a lot. She couldn't take care of herself in any way in terms of medication, food, or hygiene. Basically, taking care of herself. She would walk out at night barefoot with a nightgown, for example.*” (CF8)
	Increasing burden as caregiver	“*The medication worked less and less as the disease progressed, and it caught up with us in the end. The last three years, as I said, I had to give up, and I couldn't take care of her anymore.*” (CF4)
	Becoming a NH resident	“*He felt pain in not being at home in the apartment that he had with my grandma, his acquaintances in his community, the forest he used to walk in, all those things. I think he finds it hard that things have become distant.*” (CF6)

Dimensions of transfer quality	Receiving proper vs. insufficient medical treatment	*But he didn't get the antibiotics he needed at the hospital (…). So he got worse again the next day before he got better after that.”* (CF6)
	Obtaining information vs. being uninformed	*“That nobody has time to answer anything. It's exhausting, so to speak.”* (CF4)
	Achieving efficiency vs. long waiting hours	*“The wait. You are alone a little. You don't get anything to drink nor eat, you get nothing. You can lie for an entire day down there. You almost have to ask for yourself.”* (NHR1).

Interactions with staff members	Distinguishing between dismissive and warm and nurturing behavior	“*We need compassion. Love, basically, does a lot. (…) I think that is most important to everyone if you ask me. To show that no matter how sick you are, they love you. You want to get better… They want you to get better.*” (NHR1).
	Understanding distrust vs. trust toward staff	“*Those who are here, I trust them 100%.A handful of the nurses here are amazing. And, like I said, they have saved her life by reacting, so to speak. I think it's great. And you can say it gives me enormous comfort as a close family.*” (CF4).

## Data Availability

The data that support the findings of this study are available on request from the corresponding author. The data are not publicly available due to privacy or ethical restrictions.
